# Managing patients with eating disorders: a qualitative study in primary care

**DOI:** 10.3399/BJGPO.2024.0014

**Published:** 2024-09-18

**Authors:** Carrie Ashby, Jane Ogden

**Affiliations:** 1 School of Nutrition, University of Surrey, Guildford, UK; 2 School of Psychology, University of Surrey, Guildford, UK

**Keywords:** feeding and eating disorders, qualitative research, primary health care, general practitioners

## Abstract

**Background:**

GPs play a key role in the diagnosis and management of eating disorders (EDs).

**Aim:**

To explore GPs’ experiences of managing patients with EDs.

**Design & setting:**

A qualitative study utilising remote semi-structured interviews in the UK.

**Method:**

Fourteen GPs were interviewed about their experiences of supporting patients with EDs. The interviews were recorded, transcribed, and analysed using thematic analysis.

**Results:**

The analysis described the following four themes: (i) ‘Continuity of care’, addressing the GP's relationship with patients and family, patient transitions across life stages and geographical areas, and patient non-attendance; (ii) ‘The role of guidance’, focusing on guidelines and protocols, referrals and specialist professionals as points of contact; (iii) ‘Structural barriers’, including waiting times, lack of resources, referral criteria, and relationships between services; (iv) ‘Confidence and skills’, reflecting professional and personal experience in EDs, previous training and training needs. Transcending these themes was the notion of the ‘Limits to the care’ GPs can provide owing to professional boundaries and the emotional impact of managing patients with EDs.

**Conclusion:**

This study found that while GPs want to help patients with EDs many limits remain to the care they can provide owing to both internal and external factors. Funding is required for training and accessible specialist ED support, and greater clarity is needed regarding referral processes if ED management in primary care is to be optimised.

## How this fits in

GPs sometimes manage patients with eating disorders (EDs) yet receive little or no training. Continuity of care and guidance were key to GPs supporting patients with EDs. Patient care was undermined by many structural barriers, and GPs' own confidence and skills. Their care was also limited by professional boundaries and the emotional impact of caring for patients with EDs.

## Introduction

EDs are characterised by abnormalities in eating patterns and the nature and quantity of food consumed. Of these, anorexia nervosa (AN) has the highest mortality rate of all psychiatric illnesses, with a meta-analysis of 36 studies reporting the standardised mortality rates for AN as 5.86 and for bulimia nervosa (BN) as 1.93.^1^ Despite the morbidity and mortality associated with EDs, a study in 2018 found that most doctors trained in the UK received <2 hours of ED-related training in their undergraduate degrees.^2^ Further, the majority do not have their knowledge of EDs assessed and there is no ED training in one-fifth of medical schools.^2^ EDs present in a range of settings such as primary and secondary care, social care, and educational establishments.^3^ While much research has explored the use of different screening tools that have produced different prevalence rates, which vary according to the tool used and location,^4^ it is clear that primary care professionals play a key role in the detection of eating difficulties, assessment for any physical risks, and referral for treatment.^5^

Some research has therefore explored how GPs feel about managing EDs. For example, a qualitative study of 20 GPs from the North of England in 2010 found that although there were relatively few patients with EDs, the impact on the GP services was high owing to their complexity, lack of GP skills, and limited access to specialist services.^6^ Likewise, an online survey of Irish GPs found that more than one-third felt they did not have the skills to treat an ED, more than one-quarter reported no available resources for ED treatment, nearly half felt uninformed about local resources, the majority felt uninformed regarding ED screening, and one-third had missed an ED diagnosis.^7^ Furthermore, a survey of patients with EDs in 2021 by Beat found that 60% of patients consulting their GP felt their care was poor, 67% felt there were missed opportunities for early diagnosis and treatment, and 65% felt the care differed significantly between GPs.^8^

Therefore, while EDs often present in primary care, GPs feel unskilled and patients with EDs report that their care by GPs can be suboptimal. As a means to clarify GPs’ role in the management of EDs, a number of guidelines have been developed. These were *MARSIPAN: Management of Really Sick Patients with Anorexia Nervosa* in 2010 and 2014,^9,10^ National Institute for Health and Care Excellence (NICE) in 2017,^3^ and more recently *Managing Medical Emergencies in Eating Disorders* (MEED) in 2022.^11^ To date, however, no research has explored GPs' experiences of managing EDs in primary care since these guidelines have been published. The present qualitative study therefore aimed to explore GPs' experiences of managing patients with EDs, particularly AN, BN, and binge eating disorder (BED).

## Method

### Design

The study used a qualitative design with remote in-depth semi-structured interviews.

### Participants

Participants (*n* = 14) were recruited using a convenience sampling approach by email through the professional and social networks of the lead researcher, who has a background in general practice and ED and has worked for many years within the primary care setting. Participants were fully qualified and registered UK GPs, working in primary care with experience of working with patients with EDs.

Sixteen GPs offered to participate, but two were excluded: one GP was retired and another specialised in EDs. Fourteen GPs were interviewed.

### Procedure

Potential recruits were sent an email introducing the study together with a participant information leaflet. The purpose of the study was stated as being ‘*to explore GPs’ experiences and reflections of managing patients with eating disorders, particularly anorexia nervosa, bulimia nervosa, and binge eating disorder*’.

### Interview

In-depth semi-structured interviews were conducted remotely on Microsoft Teams, and were recorded and transcribed. The recorded interviews lasted 11–66 minutes with a mean time of 29.8 minutes. Specific questions can be seen in Table 1.

**Table 1.** Interview guide

Interview question
I would like you to consider one or more patients you have worked with who have had an eating disorder, and to reflect on their assessment and management. What do you think worked well? What worked less well? What did you find helpful? What was less helpful? Can you think of anything that may have helped you look after the patient(s)? How confident did you feel in managing the patient(s)? Are you aware of any local or national guidelines relevant to eating disorders? Do you have any knowledge about these guidelines? Are you aware of any services or resources that may be helpful? Have you had any training or education in eating disorders? Was this as a medical student, doctor in training, or since qualifying as a GP? Would you be interested in receiving further training? If so, what type of training might be helpful? Do you have any comments on the support you or the patient received from other healthcare professionals or services? Do you have any other reflections on managing patients with eating disorders? Do you have any further comments?

### Data analysis

The data were analysed manually using thematic analysis, which incorporates the following six phases: familiarisation with the data; code generation; theme generation; review of themes; defining and naming themes; and report production.^12^ The analysis was discussed within the research team and a thematic map was developed in an iterative manner. All interviews were conducted by one female researcher, a medical doctor who has worked in general practice and an eating disorders service (EDS). All participants were female GPs, some of whom knew the researcher professionally and/or personally before the study. These factors may have influenced the interview and could have either made participants more open in their answers owing to a shared professional status or less open if they felt that they were being judged. To mitigate against any sense of judgement, the researcher emphasised that they were there in a research capacity and were personally aware of the many difficulties facing GPs trying to support their patients with EDs.

## Results

### Participant demographics

All 14 participants were female, identified as White British, had been qualified GPs for 3–30 years, and worked across the UK (see Table 2).

**Table 2.** Participant demographics

Pseudonym	Years of GP qualification	Geographical area of work	GP role	Sex	Ethnicity
Fiona	20	Hampshire	Retainer GP Former partner	Female	White British
Jane	18	Southampton	Salaried GP	Female	White British
Julia	23	Hampshire	GP partner	Female	White British
Megan	30	Shropshire	Locum GP Former partner	Female	White British
Hannah	22	Thames Valley	Salaried GP	Female	White British
Debbie	22	South Wales	Locum GP Obesity medicine physician	Female	White British
Tanya	3	Thames Valley	Salaried GP	Female	White British
Tricia	29	Hampshire	GP partner	Female	White British
Emma	7	Hampshire	Locum GP	Female	White British
Charlotte	6	Hampshire	Salaried GP	Female	White British
Claire	22	Northeast England	Locum GP Former salaried GP	Female	White British
Lisa	10	Dorset and Hampshire	Locum GP Former salaried GP	Female	White British
Maggie	10	Fife	Academic GP Locum GP	Female	White British
Jo	20	Hampshire	Salaried GP	Female	White British

### Thematic analysis

**Figure 1.**
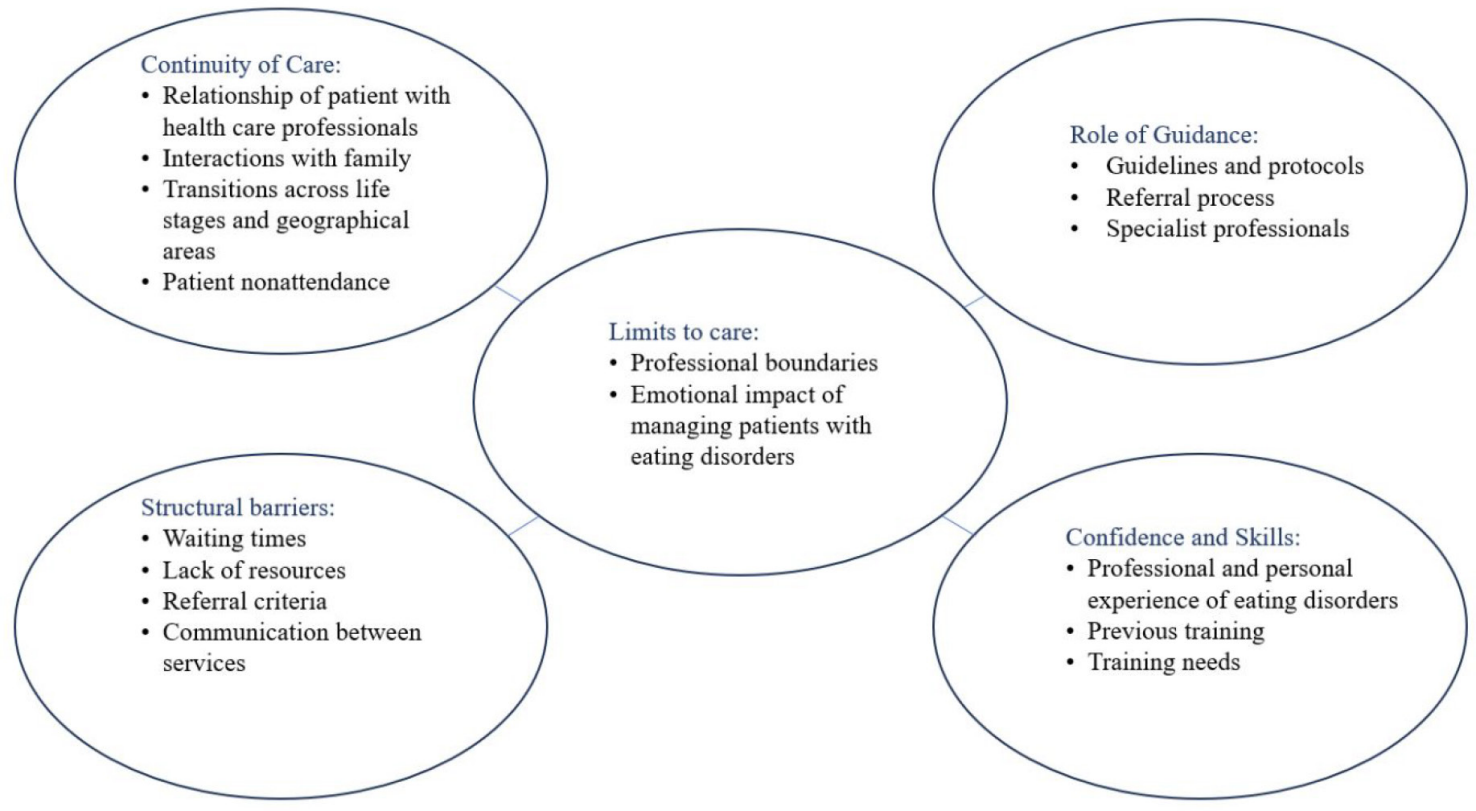
Thematic map

Manual thematic analysis led to the description of four main themes with a transcending theme (see Figure 1).

#### Theme 1: The value of continuity of care

Several GPs cited continuity of care as helpful in the management of EDs with a focus on relationships of professionals with patients, ongoing conversations with families, transitions across life stages and geographical locations, and patient non-attendance.

##### Relationship of patient with healthcare professionals

Several GPs said that establishing a good relationship was key for patient care. For example, Emma said:

*‘What went well is that I worked at the same surgery for six years and managed to get a rapport with her, so we had some continuity going … we understood each other, and I knew when she was getting worse and when she was getting better.’* (Emma)

Similarly, Megan expressed how everyone also benefits when the patient can build a good relationship with a specialist colleague, which can help the patient, their families, and GPs:

*‘It makes a difference to us, it means a lot … they’ve got the nice person that they like, who likes them, it’s huge, being held. The fact that they’ll attend is massively supportive for all the parents, husbands, I suppose for the doctors as well.’* (Megan)

##### Interactions with family

GPs also described the importance of involvement from family members, which in some cases was instrumental to diagnosis:

*‘She was telling me absolutely everything was fine, but as she left her Mum … said "That’s not the case, this is wrong, she’s really unwell, she’s not eaten for a week"; she obviously had to go to A&E.’* (Emma)

GPs also commented how reassuring it was for them to know that families are involved:

*‘Another positive thing is having parental input and support, so often they’ve attended with a parent … as a general practitioner it’s probably peace of mind knowing that you’ve got a parent involved and aware of the situation.’* (Charlotte)

##### Transitions across life stages and geographic locations

Maintaining continuity of care was not always easy, however, and could be disrupted. Sometimes this was through stages of transition that could present a risk to patient management such as returning home in university holidays, which was seen as a time of heightened risk:

*‘It feels really dangerous because obviously it’s a huge change. It’s lovely that they’re going home but they’re getting out of routine. It’s when they’re probably at most risk of things going pear-shaped.’* (Julia)

Several GPs also described how patients transitioning from paediatric to adult services can bring challenges, owing to issues surrounding consent and confidentiality, differences between paediatric and adult services, and a break in continuity of care:

*‘When she was a child, it was easier to get help and the issue of consent wasn’t such an issue because Mum would come to appointments, and she had a good relationship with her Mum. When I rang Paeds* [Paediatrics]*, the conversation would go much smoother than when I rang the adult services and spoke to the Med Reg* [Medical Registrar]*.’* (Lisa)

Patients joining a practice from overseas can also be a difficult transition:

‘*There was a discharge letter from her Australian GP which said that two weeks prior the patient had been in ITU* [Intensive Treatment Unit] *with a potassium of 2.7, purges four or five times a day, and you get half the notes because she’s not been in the country.’ (Emma*)

##### Patient non-attendance

GPs also described how continuity of care could be challenged through the prospect of being weighed, which could deter patients from attending appointments:

*‘Being asked to stand on the scales is often the only thing they’re thinking about until it’s happened, and it can still dominate the whole consultation yet it can be a real barrier to either turning up or engaging in anything else in the consultation.’* (Jo)

In summary, this first theme highlights how GPs valued continuity of care, which can facilitate a good rapport between healthcare professionals, patients, and their families. In contrast however, non-attendance and transitions through different life stages, different services, and geographical areas can adversely affect continuity of care and impact on their care of those with EDs.

### Theme 2: Role of guidance

Participants discussed the value of guidance in managing patients with an ED with a focus on guidelines and protocols, referral forms, and contact with specialist professionals.

#### Guidelines and protocols

Most of the GPs said they refer to guidelines when looking after patients with EDs. Fiona described how guidelines help her:

‘*Having those* [guidelines] *somewhere in the back of your head or thinking about what kind of examination, tests you need to order so you’re ready prepared for what you might be looking for … I might just quickly scan them before they come in.’* (Fiona)

As patients may consult all members of the practice team, Jo also said it was very helpful for primary care staff to have good guidelines:

‘*Clear guidance on what we’re looking for, and what would trigger further input ... what things should be worrying or unnerving to a GP or potentially a healthcare assistant or somebody who wouldn’t know what to spot within the practice …. please contact us if they’re like this, we’re very worried, please contact us immediately.’* (Jo)

#### Referral process

Most of the GPs said a referral form could be useful:

*‘We have proformas that you fill out and they’re much better than they used to be because there’s lots of tick box things so I can check I’ve done all the required investigations before I send the referral.’* (Hannah)

Despite this, the process of referral forms was also a source of irritation:

*‘Form-filling can feel a bit annoying but there are some advantages to a form because it triggers you to put in the information that the person really needs ... I think my preference is a form with a big free-text option.’* (Jo)

#### Specialist professionals as a point of contact

Several GPs commented on the importance of being able to obtain specialist advice:

*‘As a GP, knowing that there’s somewhere else to go if the situation becomes unmanageable in primary care … knowing where the back-up is and it being reliable and being able to use it and communicate with it easily is probably what I would find most useful.’* (Jo)

This support wasn’t always there, however, and Debbie said she felt unsupported by her local EDS:

‘*It’s been really poor ... the adult eating disorder service was almost non-existent, and I really felt unsupported. I could never get hold of anybody.’* (Debbie)

Some GPs suggested that it may help to develop lead roles for EDs within primary care:

*‘If you’re really wanting to upskill GPs that they would see all the eating disorders in a practice … there’s so much knowledge accumulation all the time that we can’t all be experts in everything.’* (Maggie)

Megan thought practice nurses or healthcare assistants (HCAs) could also take a lead role:

*‘It’s possible it’s saying something like is there a practice nurse in each practice that would lead this ... or even a healthcare assistant who would buy in to it and that would work’* (Megan)

To summarise, most of the GPs said they found guidelines and protocols useful for supporting patients with EDs, including prompts on referrals. Several commented on the importance of access to specialist advice, some expressed a need for case managers or specialist nurses and some felt it could be helpful to have lead staff for EDs in primary care.

### Theme 3: Structural barriers

The GPs discussed many structural barriers to their care of those with EDs, including waiting times, referral criteria, relationships with specialist services, commissioning issues, and lack of resources.

#### Waiting times

One barrier to supporting those with EDs was long waiting times:

*‘One of the bugbears that GPs have is there’s such a long wait for these patients to be seen … leaving us holding the risk because we end up doing the monitoring, don’t we … and that can feel worrying for the GP.’* (Hannah)

Although GPs were satisfied once the specialist treatment had started:

*‘The people who did get into our local service and have support were really happy with it … our local counsellors were excellent, they’re very talented clinicians because it’s a really, really difficult subject, it’s just getting them in.’* (Emma)

#### Lack of resources

Lack of resources also presented a problem for optimal care of those with EDs. For some GPs, this related to the expectation of them carrying out checks they are not paid to do:

*‘They are requesting the GP to do weekly weights and a healthcare assistant assessment, probably worth 20 minutes of their time, and my understanding is general practice is not commissioned to undertake this … you had secondary care saying "you need to do the physical health monitoring" and then you have the GP surgery saying "no no no we’re not doing the physical health monitoring, we’re not commissioned to do that."’* (Charlotte)

Likewise, this lack of resources was also reflected in problems with specialist services:

*‘I can make a referral, but I don’t know how much they can really do because they’re fighting fires with the really sick anorexics* [sic]*.’* (Tanya)

#### Referral criteria

Several GPs also felt that the focus on weight presented a structural barrier to care. At times this was owing to the need for a low weight to get a referral:

*‘It’s terrifying that we can’t get people into the eating disorder service unless they have a low BMI* [body mass index]*. I think we’re missing vast numbers of people who could benefit from help.’* (Tanya)

But this barrier to care also related to the role of weight gain as part of the definition of engagement or the failure to engage. For Charlotte this felt unjustified:

‘*She didn’t want to put on weight or to start eating … I think they felt she wouldn’t engage and discharged her but I was like surely this is the person that needs help more than the person that’s willing to engage.’* (Charlotte).

This could also lead to referrals being rejected, which was frustrating for GPs:

*‘Someone saying no is always tricky for me and patients, so I think that sense of not having met criteria or there being a barrier to care is always unhelpful.’* (Jo)

#### Communication between services

The final structural barrier related to communication between services. One GP described a negative experience after referring someone to her local EDS:

*‘We had a big, long discussion about how she needed to go and see the consultant who was running the eating disorders service and when she got there, she drank 3 litres of water before her weigh in. So, he wrote me a nasty letter back saying, "she’s a normal weight, why did you bother to refer this woman?"’* (Debbie)

At times, specialist services may also disagree on responsibilities:

*‘There were quite a few tussles, I’d see quite a lot of interaction … who took them on, especially with the children. There was a bit of a fight between them.’* (Emma)

Julia also expressed frustration with the sharing of blood results:

*‘It’s frustrating there’s no national system, of being able to just deposit blood tests and that’s where with the NHS App you’d have thought now that the fact the patients got them on an app they would be able to do something with that information and send it themselves’.* (Julia)

In contrast, however, most GPs generally felt EDS correspondence was good:

*‘They’re good at letting you know they’ve been in touch with the patient, that they’re booking the patient an appointment, and also keeping you up to date with progress and when they’ve finished.’* (Jane)

In summary, the GPs described many structural factors that got in the way of caring for patients with EDs including waiting times, the referral process, and criteria with its focus on weight, lack of resources, and poor communication.

### Theme 4: Confidence and skills

This final main theme reflects the role of GPs’ confidence and skills in managing patients with EDs, and how this was influenced by professional or personal exposure to EDs and training.

#### Professional or personal experience of eating disorders

The GPs described how their confidence and skills in managing patients with EDs were closely related to their exposure to the condition, which was often low as patients with EDs are not that common in primary care. For example, Hannah described how she felt:

*’Fairly unconfident I would say because they come round quite rarely for the GP’.* (Hannah)

When they had had some professional exposure to those with EDs, however, they felt much more comfortable in supporting their patients. As Lisa said:

*‘I feel more confident because I’ve done an eating disorders inpatient job as a junior doctor ... I’m happy looking at the QTc interval* [on ECGs] *and what I’m looking for, because of my experience of working on the unit.’* (Lisa)

Personal experience was also key to gaining confidence with managing EDs. For example, Tanya described how they had been deeply affected by their friend’s illness:

*‘One of my best friends at school ended up in* [an EDU] *for 2 years in total with anorexia and I got to know various people with eating disorders ... I feel that because I have a bit more than average knowledge about it from the personal side, I feel I’m more able to help them.’* (Tanya)

#### Previous training

Previous training in EDs was also related to their sense of confidence. Five GPs had never received any EDs training and most of the others received minimal training, including as GP registrars:

*‘No, it was not really mentioned, it’s kind of an awareness for me because I’ve got a couple of friends with eating disorders, so it has logged in my memory that it was pretty poor’.* (Lisa)

*‘On my VTS* [Vocational Training Scheme] *I can honestly say I had zero training’.* (Hannah)

One of the GPs recalled attending a course where a doctor with lived experience gave a talk:

*‘With one of our study days we had actually a junior doctor who’d had anorexia and was giving her own view on what it was like to be on the other side, to be a patient and things.’* (Tricia)

#### Training needs

Given their absence of training, most of the GPs said they would be interested in more training, *‘it would be really good to know that I was doing the right thing with my small window of opportunity’* (Tanya), although Fiona acknowledged the competing demands on study time: *‘It’s always an important area ... however there’s so many demands on education, you name the subject, and I could go "of course, I need more education in that".’*

The GPs listed a number of topics they would like to learn more about including diagnosis and assessment (Claire), managing those on waiting lists, the use of vitamins, bone scans, and anti-depressants (Hannah), what to cover in the first consultation in terms of safety features (Tanya), signposting and conversations with parents, weight measurement (Megan), free resources (Emma), and interactive case studies (Maggie).

In summary, previous professional and personal experience can improve confidence in managing EDs. Most of the GPs had received little formal education in EDs and were interested in further learning.

### Transcending theme: Limits to care

GPs therefore talked about managing patients with ED in terms of the role of continuity of care, the use of guidelines, structural barriers, and their confidence and skills. Transcending these themes was the notion that there are limits to the care GPs can provide for patients with EDs owing to professional boundaries and the emotional impact of caring for patients with EDs.

#### Professional boundaries

While GPs wanted to help patients with EDs they recognised the scope of their practice and the limits to the care they can provide. As Debbie said:

*‘I’m happy to follow the guidelines and do the relevant blood tests but I don’t think I’m the right person to be managing someone with an eating disorder. I think they need specialist help so as far as I’m concerned, my job is signposting to secondary care.’* (Debbie)

Fiona also felt her role was to signpost:

*‘I’m not a specialist. My job is to know is there a problem and do I need a specialist and it’s not to be the specialist, it’s not to fix it, it’s to recognise and to signpost.’* (Fiona)

Similarly, Tanya also acknowledged she is not a specialist but still wanted to be able to support her patients:

*‘I’m not a therapist, so I can’t do therapy, but at the same time I should be able to say the right kind of encouraging things.’* (Tanya)

#### Emotional impact of managing patients with EDs

In addition to professional boundaries, GPs also described how managing EDs can be difficult. As Maggie said:

*‘It’s challenging and it’s emotive and it’s worrying and its difficult.’* (Maggie)

One GP described the anxiety of managing an unwell patient:

*‘I felt very unsafe, mainly because I felt I was working by myself with such an acutely unwell lady who really wasn’t playing the game ... that was a very difficult experience actually.’* (Emma)

For some, EDs were particularly difficult to comprehend owing to their own feelings about food and their own sense of self:

*‘It’s not an area that I’m particularly fond of treating because I find it really difficult and I don’t understand because I love food, so I kind of don’t have that personal ability to relate … I feel a bit out of my depth. How do you help people when they are so lost in this belief and hatred of themselves?’* (Fiona)

For some GPs, however, while emotionally difficult, managing EDs can also be rewarding:

*‘I think it’s a challenge, I think it’s time-consuming but on the other hand, it’s quite rewarding when you see someone coming out the other end of it.’* (Julia)

In summary, overarching the four themes of continuity of care, guidance, structural barriers, and confidence and skills was the transcending theme that there are limits to the care GPs can provide, owing to their professional boundaries and the emotional impact of caring for patients with EDs.

## Discussion

### Summary

This study aimed to explore GPs’ experiences of managing patients with EDs, particularly AN, BN, and BED. Primarily, GPs emphasised continuity of care as key to being able to offer appropriate care and felt that good relationships between themselves, the patient, and often their families were paramount. Such continuity, however, could be disrupted by movements between child and adult services; or when the patient moved away from home to study; or patient non-attendance was sometimes triggered by weighing. Second, GPs highlighted a role for guidelines, protocols, and referral forms as helpful and felt that it was important to be able to access specialist advice quickly, particularly in an emergency. Such structural support was not without its problems, however, and could be confusing or time-consuming. Third, GPs described structural barriers to patient management including waiting times, inadequate resources, referral criteria, and communication between services. Finally, the interviews addressed confidence and skills, with sub-themes relating to professional and personal experience of EDs, previous training, and training needs. Transcending these themes was a description of the limits to the care GPs can provide owing to a respect for professional boundaries and the emotional burden experienced when managing patients with EDs.

### Strengths and limitations

The study provides an in-depth account of how GPs experience managing patients with EDs. There are some problems, however, that need to be considered. First, the study sample were all female and described themselves as White British. While EDs still affect more female patients and while female patients tend to select female GPs to discuss EDs, further research is needed with a wider sample to explore a more heterogenous set of experiences. Furthermore, all GPs implicitly expressed an interest in managing patients with ED, as illustrated by their willingness to participate in the study. Their willingness to engage in training about EDs may not reflect the views of those less interested in ED care.

### Comparison with existing literature

The focus on continuity of care as core to managing patients with EDs reflects previous research across many other conditions,^13,14^ which can promote help seeking^15^ earlier diagnosis and quicker referral.^16^ It also supports the power of a good relationship with just ‘one specialist professional’ if this is someone they could trust and build a therapeutic relationship with.^17^ Further, it highlights how problems with communication and issues of confidentiality can arise during transitional periods, which need careful management.^10,18,19^ The GPs also spoke about the role of guidelines, reflecting the range of guidelines that have been developed to support GPs. The results provide some support for this approach but illustrate that, in line with previous studies, such interventions do not come without their problems.^20,21^ Access to rapid telephone or email advice, which can reduce written referrals and hospital admissions, may be more useful.^20^

The role of structural barriers to the effective management of those with EDs also reflects earlier research in which GPs expressed frustration with long waiting times leading to patients’ needs not being met.^6^ It also parallels the ongoing need for more EDSs, understaffing, inadequate funding, and lack of training.^17,22,23^ The focus on body mass index (BMI) was specifically highlighted as a barrier to care, which is consistent with previous research showing patients with EDs with higher BMIs are frequently underdiagnosed, undertreated, and excluded from treatment.^24^ Further, poor communication between primary and secondary care was also seen to be problematic, reflecting a call for a more multidisciplinary shared-care approach from diagnosis through recovery.^17,22^

GPs in the present study also described their lack of confidence and skills as a barrier to managing patients with EDs.^23^ They were receptive to further training^17^ and listed areas they wanted training in: these included diagnosis, assessment, medical emergencies, vitamin supplements, bone health, medication, refeeding, how to weigh patients, resources for patients and carers, and interactive case studies. Some were also supportive of appointing lead ED clinicians in primary care to improve continuity of care.^14^

Transcending these themes was a description of the limits to the care GPs can provide. First, this was owing to a respect for professional boundaries and a sense that although they wanted to help their patients with EDs, they needed to consider the scope of their practice. This reflects previous research in which GPs said they could be sympathetic and supportive to patients with EDs, but felt they lacked the necessary expertise and therefore preferred to refer patients for treatment.^5^ This also reflects GPs’ experiences of managing other conditions they feel are beyond their levels of expertise. For example, Epstein and Ogden^25^ interviewed GPs about the management of obesity; the GPs said that while they could listen to the patient, they did not feel that obesity management was within their domain.

Second, GPs also discussed the limits of care they could provide in terms of the emotional impact of managing patients with EDs, describing it as ‘*worrying*’, ‘*difficult*’, ‘*emotive*’, ‘*a challenge*’, and ‘*terrifying*’. In 2010, Reid and colleagues described how GPs found the management of EDs ‘*frustrating*’, ‘*difficult*’ and an ‘*awful lot of work*’.^6^ Thirteen years later it would seem that not much has changed.

### Implications for research and practice

The findings from the present study have implications for research and practice. In terms of research, future studies could investigate the impact of patients regularly seeing the same staff members or having lead ED clinicians in primary care, and clear guidance on referrals and management. Further, it would be useful to assess whether quicker access to specialist advice reduces hospital admissions and referrals. It would also be useful to establish whether GP training, as outlined by the GPs in the present study, improved both their own confidence and skills, and whether in turn this could improve patients’ satisfaction with their care.

In terms of practice, the results highlight many areas for improvement. In particular, given the key role of continuity of care, a lead GP and nurse for EDs could be introduced for each practice. Further, all medical students and doctors could receive relevant training in EDs; services need to agree and disseminate clear guidelines and referral forms, with an agreed strategy for non-engaging patients, and GPs should be informed who a patient’s case manager is and how to obtain advice from specialist services. All of these require improved resourcing for EDSs.

To conclude, four themes were described from interviews with 14 GPs about their management of patients with EDs, relating to the importance of continuity of care, the role of guidance, the impact of many structural barriers, and problems with their own confidence and skills. Further, the interviews highlighted limits to the care GPs can provide, owing to professional boundaries and the emotional impact of caring for patients with EDs. Therefore, while GPs may be motivated to support patients with EDs, the level of care they can provide is often undermined by both external and internal factors. Accordingly, much more is needed to improve both the clinical environmental surrounding this care and the GP’s own knowledge and skills with this patient population.

## Data

Due to the potential for identification, the GPs did not consent for the interview transcripts to be made publicly available.
